# Orphan proteins of unknown function in the mitochondrial intermembrane space proteome: New pathways and metabolic cross-talk

**DOI:** 10.1016/j.bbamcr.2016.07.004

**Published:** 2016-11

**Authors:** Esther Nuebel, Phanee Manganas, Kostas Tokatlidis

**Affiliations:** Institute of Molecular, Cell and Systems Biology, College of Medical, Veterinary and Life Sciences, University of Glasgow, UK

**Keywords:** Mitochondria, Protein import, Intermembrane space, Redox signalling, Metabolism, Mitochondrial proteome

## Abstract

The mitochondrial intermembrane space (IMS) is involved in protein transport, lipid homeostasis and metal ion exchange, while further acting in signalling pathways such as apoptosis. Regulation of these processes involves protein modifications, as well as stress-induced import or release of proteins and other signalling molecules. Even though the IMS is the smallest sub-compartment of mitochondria, its redox state seems to be tightly regulated. However, the way in which this compartment participates in the cross-talk between the multiple organelles and the cytosol is far from understood. Here we focus on newly identified IMS proteins that may represent future challenges in mitochondrial research. We present an overview of the import pathways, the recently discovered new components of the IMS proteome and how these relate to key aspects of cell signalling and progress made in stem cell and cancer research.

## Highlights

• A brief overview of the classic mitochondrial import pathways is featured
• Recent studies assigning a number of new proteins to the mitochondrial IMS are discussed
• Analysis of the expanded IMS proteomes can provide insights into organelle cross-talk and signalling pathways

## Graphical abstract

**Figure:**
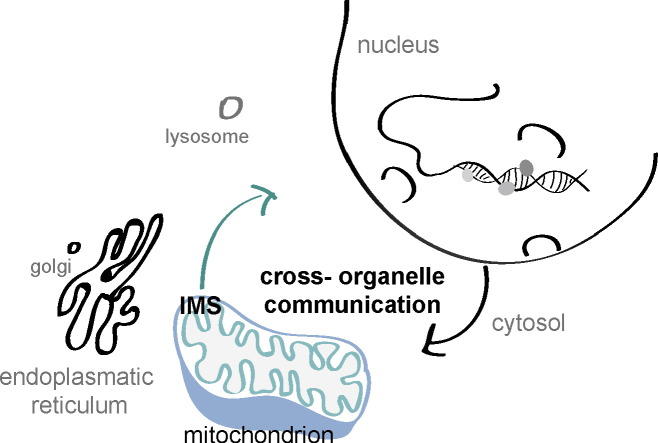

## 1 Introduction

Mitochondria are traditionally described in biochemistry textbooks as the power plants of a cell; although this is true, this limiting description underestimates the far reaching roles of this organelle in a variety of critical cellular processes unrelated to energy production. Mitochondria are key players in several cellular processes, including respiration, biosynthesis, apoptosis signalling and ion homeostasis. They are also generators of reactive oxygen species (ROS) which are themselves players in different signalling pathways [1], [2]. These pathways share specific redox reactions [3], in which the mitochondrial oxidative defense system [4] contributes to maintain redox homeostasis.

Mitochondria have been implicated in several diseases, such as Parkinson's and Alzheimer's [5], [6]. Additionally, their involvement in cancer [7], [8], apoptosis signalling [9] and stem cell development [10] is under extensive research.

Modern techniques enabled the investigation of the internal structure and morphology of mitochondria and revealed a highly complex compartmentalisation [11], [12]. The organelle is surrounded by a double membrane. This allows the assignment of the mitochondrial matrix (MM), the inner membrane (IM) the intermembrane space (IMS) and the outer membrane (OM). The inner membrane forms characteristic invaginations called cristae, which form an additional specific environment since they are separated from the inner boundary membrane by cristae junctions. Each different biosynthetic pathway can be assigned to a specific compartment, but the challenge is to dissect the communication and maintenance of the individual compartments. Part of this maintenance is to ensure proteostasis (folding, unfolding and degradation) to generate a homeostasis of the functional proteome and to clear mistargeted and damaged proteins. Every sub-mitochondrial compartment needs to control its redox milieu, which is interestingly highly different. The inner membrane separates the reducing matrix from the more oxidizing IMS [13]. For a long period of time, researchers focused on investigating the import pathways of the hundreds of nuclear encoded mitochondrial proteins. This culminated in the discovery of the major pathways that translocate proteins through the dedicated translocon complexes of the outer membrane (TOM), the inner membrane (TIM), as well as the MIA (Mitochondrial intermembrane space Import and Assembly) pathway, for the proteins that are targeted to the intermembrane space [14], [15], [16], [17]. One of the big challenges of future research will be to investigate how the organelle communicates with the cytosol and the nucleus. The IMS with its specific redox environment and the ‘controlled leakiness’ of the outer membrane due to the presence of porins that allow the free diffusion of molecules less than 5 kDa, might harbour candidates which mediate the communication from signalling occurring inside the mitochondria towards other organelles, the cytosol and the nucleus. Of particular interest in this respect are newly identified IMS-resident proteins of known and unknown function and proteins with at least a dual localisation. For simplicity these proteins will be named ‘orphans’ not with the intension to classify necessarily their function as unknown but paying tribute to the fact that they have been assigned to the intermembrane space of mitochondria only recently. Such proteins could lead us to investigate new routes in mitochondrial research. In this review we aim to investigate new identified orphan proteins that are either soluble in the IMS or associated with the inner membrane and have a functional domain in the IMS. We interpret their features to address the possible pathways they might be involved in. This perspective will be complemented by an analysis of how signalling via metabolites and epigenetic modification may contribute to the intercellular cross-talk.

## 2 Mitochondrial import

Mitochondrial import has been studied extensively over the last decades [14], [18], [19], [20], [21], [22], [23], [24], [25], [26]. There is still potential to unravel new pathways in addition to the well-established import routes. Protein import normally starts with binding of chaperones to the precursor located in the cytosol, followed by binding to import receptors located at the outer mitochondrial membrane. The precursors then pass through the TOM complex, which, in yeast, is made up by the proteins Tom20, 22, 70, the pore forming Tom40 protein and small components named Tom 5, 6, and 7. The precursor is subsequently guided via the TIM receptors Tim50 and Tim23 and engages the TIM pore which is formed by the Tim17 and 23 proteins. The import process of matrix proteins is driven to completion by an ATP consuming step, which involves Tim44, mitochondrial Hsp70 and the nucleotide exchange factor GrpE (Mge1). If a targeting signal is present, this is cleaved by an internal protease, which subsequently allows the refolding of the protein [21], [27]. The sorting and assembly (SAM) pathway for outer membrane proteins as well as the second TIM pathway and the MIA dependent import into the IMS are distinct translocons that allow a compartment-specific localisation of nuclear encoded proteins.

## 3 Import into the IMS

Here, we focus on two main pathways for import into the IMS which have been well characterised. The first class of proteins follows the same import route as matrix proteins but a hydrophobic so called “stop-transfer” sequence causes an arrest of the precursor within the TIM23 complex, cleavage of the N-terminal presequence peptide by the matrix peptidase MPP, followed by a second proteolytic cleavage of the stop-transfer sequence in the IMS and release of the mature protein into this compartment [28]. Different peptidases are known to participate in this second cleavage event depending on the substrate protein, e.g. cytochrome b_2_ and Mgm1 are cleaved by Imp1 and Pcp1, respectively. A second class of IMS proteins are the ones that acquire disulfide bonds during their biogenesis. A dedicated machinery oxidizes these proteins as part of their import process into the IMS [29], [30], [31], [32], [33]. As mentioned earlier, the redox status of the IMS is oxidizing and is required for this pathway. It is likely that the IMS of mitochondria has maintained the oxidizing environment of its corresponding bacterial compartment, the periplasm, during the endosymbiotic process. The redox potential of the intermembrane space is around − 225 mV, which is more oxidizing than that of the cytosol at − 290 mV [13]. However, Kojer and coworkers [34]suggested (using a new generation of roGFP-based redox-sensors) that the levels of GSH are maintained in the IMS through a porin-mediated diffusion from the cytosol. The identification of the enzymes, Mia40 [30] and Erv1, which perform regulated transfer of disulfide bonds to substrate proteins, was a substantial advance for the intermembrane space oxidative pathway [35].

The mechanism for protein import into the mitochondrial intermembrane space starts with the reduced and, therefore, disulfide bond free substrates entering via the OM. Disulfide bonds are introduced by Mia40, and electrons are transferred from Mia40, to oxidized Erv1, and finally to oxygen produced by the respiratory chain via cytochrome c and complex IV [36]. The final electron acceptor under anaerobic conditions remains elusive. The introduction of a disulfide bond increases the tertiary structure of the substrate and therefore traps the protein in the IMS.

The targeting sequence for this class of proteins is called Intermembrane space Targeting Signal (ITS) or mitochondrial IMS-sorting signal (MISS) and is found additionally to the CX_n_C cysteine motifs (e.g. the CX_9_C motif in yCox17) [37], [38]. The ITS is characterised by some key properties: (i) it can function upstream or downstream of the cysteine which interacts with Mia40, also called the docking cysteine; (ii) it is sufficient for crossing the outer membrane and even for targeting non mitochondrial proteins; (iii) it forms an amphipathic helix with hydrophobic residues facing the side of the docking cysteine and dispensable charged residues on the other side and (iv) its fit is complementary to the substrate cleft of Mia40 via hydrophobic interactions [39].

## 4 Proteome of the IMS

The complexity of the proteome supersedes that of the genome, due to alternative splicing events and post-translational modifications, such as phosphorylation or methylation. Here, we want to focus on the recently undertaken approaches to investigate the proteome of the smallest sub compartment of mitochondria, the intermembrane space. The intermembrane space proteome requires careful analysis since the last decades of research have pointed out the importance of this sub compartment in processes such as (i) protein transport, (ii) lipid transport, (iii) regulation and assembly of the respiratory chain, (iv) regulation of redox processes, (v) coordination of apoptosis and (vi) metal homeostasis. It is under investigation to unravel the so far unknown pathways the IMS proteome might be involved in [40]. The investigation of the IMS holds potential to unravel players which are involved in cysteine oxidation due to its oxidizing environment, unlike the prevention of cysteinereduction in other cell compartments like the cytosol [41]. It is also tempting to speculate about the presence of a very tightly regulated redox sensing mechanism which will help to adjust the metabolism to a given stressor. In cases of stress, induced from a variety of sources including chemicals, metals, or particular diseases, the cell needs to make the decision whether to undergo programmed cell death or repair. In the case of apoptosis, it is widely recognised that the IMS releases part of its proteome [42]. The molecules which might eventually be targeted to the IMS only under specific stress conditions have not yet been investigated in detail. Previous studies could identify and verify the localisation of 31 IMS proteins in yeast [43] and 23 IMS proteins in human mitochondria [44]. More recent investigations have been able to detect a number of new IMS orphans in both organisms. More specifically, these efforts were performed using the established yeast model system *S. cerevisiae*[43] and, more recently, human cells. Hung and co-workers were able to extend the current human IMS proteome to a protein count of 127 [45].

## 5 New identified orphans of the IMS

The main challenge in proteomics is to achieve high purity of subcellular regions without contaminations from distinct organelles. This is not a trivial task, as contact sites between organelles are frequently part of their normal function and separating them in homogeneous and pure fractions is very difficult. Since this technical challenge limits our understanding of pathways and mechanisms in living cells, researchers have devoted substantial efforts to alleviate this problem. In the recent literature, two attempts have been reported to create a full inventory of the IMS proteome. Vögtle et al. induced BAX mediated release of the IMS proteome to map all IMS located candidates in *S. cerevisiae* cells [43]. More recently, another group developed a very intriguing method, where they used an engineered ascorbate peroxidase (APEX), which was genetically targeted to the IMS. Upon addition of biotin-phenol and hydrogen peroxide (H_2_O_2_), APEX covalently tags surrounding endogenous proteins with the biotin-phenoxyl radical oxidation product. Cells can be lysed and biotinylated proteins get separated using streptavidin beads and mass spectrometry follows for protein identification. In combination with a stable isotope labeling by amino acids in cell culture (SILAC)-based ratiometric tagging strategy, allowed these authors a very accurate and extensive insight into the IMS proteome from HEK293T cells. In this study, the putative outer membrane proteome and new orphans established as real IMS proteins were reported [45]. Here, we investigated reported orphans from both studies. We believe that analysing the whole set of new identified IMS candidate proteins will allow to cluster them in different pathways and therefore design new strategies on how mitochondrial research could be focussing in the future. The detailed features of these newly identified IMS proteins (37 in human and 20 in yeast) is compiled in Table 1. These proteins are listed as new IMS proteins despite the fact that some have been reported to localize in mitochondria before, but not specifically in the IMS. Also, proteins which are strongly associated or even inserted into the inner membrane but display their function in the IMS compartment are considered as IMS protein in this review. The table displays databank entry, gene name, protein name, as well as mitochondrial localisation prediction and presence of cysteine motifs (used databases are mentioned in detail in Table 1).

Based on experimental evidence and text-mining of available literature, we were able to assign some of the candidates into specific subgroups which will be discussed in the following chapters.

## 6 Recently discovered orphans tested for stop transfer IMS import

As mentioned earlier, some intermembrane space precursors harbour sequences that function as putative stop-transfer domains to arrest translocation of proteins during import and target the precursor to the intermembrane space. yPtc5 (YOR090C), a mitochondrial type 2C protein phosphatase, is plausibly presented from Vögtle et al. to follow the presequence pathway and subsequently be processed by an inner membrane protease in order to be finally released to the IMS [43]. Previously, this protein was suggested to be involved in the regulation of the pyruvate dehydrogenase complex by dephosphorylating the serine 133 of the yPda1p subunit [46]. This complex is located in the mitochondrial matrix, whereas the branched-chain α-ketoacid dehydrogenase complex (BCKDC) which is cross reacting with PDH complex proteins, is located on the inner mitochondrial membrane. As now the protein has been convincingly located in the IMS, an interaction with a complex in the inner membrane seems more likely. A deficiency in enzymes of the BCKDC complex or its inhibition lead to accumulation of branched-chain amino acid and their derivatives, called ketone bodies. Ketone bodies cause a sweet smell to bodily excretions and lead to a pathology known as maple syrup disease. Assigning yPtc5 to the IMS provides the opportunity to investigate if the BCKDC complex might be regulated via yPtc5 mediated dephosphorylation and thus opens new perspectives in maple syrup disease research.

## 7 Recently discovered orphans tested for MIA40-dependent import

MIA-dependent precursors typically contain twin CX_9_C or CX_3_C motifs [39]. In the list of new identified orphans, only two of the proteins display this characteristic, yPet191 and yCmc2. Interestingly, yPet191 carries the typical cysteine motif but seems to be imported independently from the MIA pathway [47]. However, other proteins with uncommon cysteine motifs (see Table 1) could be identified, including yCox12, Ymr244c-a (yCoa6), yNce103 and Ybl107c (yMix23). The results from Vögtle and co-workers demonstrate that the proteins with uncommon cysteine motifs, except yNce103, require the MIA import machinery [43]. The yNce103 protein was not further investigated. Furthermore, another set of MIA-dependent precursors, which do not require their cysteine motifs in order to become imported into the mitochondria, has also been identified. This group includes the yeast proteins yAtp23 [48], yTim22 [49], yMrp10 [50] and the human protein CHCHD3 [51]. Therefore, every newly discovered orphan could also be a MIA substrate, although this would need to be tested experimentally.

Another important aspect concerns quality control systems of newly imported IMS proteins. Therefore, the identification of prolyl 4-hydroxylase (hP4HB), a multifunctional enzyme that belongs to the protein disulfide isomerase family, might be a first indication of the presence of such a control system in the IMS. This enzyme also displays disulfide isomerase activity. Based on the presence of two thioredoxin domains which can catalyze the formation, breakage and rearrangement of disulfide bonds, this protein might play a role in overcoming the aggregation of misfolded proteins [52].

## 8 IMS candidates involved in mitochondrial respiration

Ymr244c (yCoa6) is a protein involved in cytochrome c oxidase (Complex IV) assembly and achieves the delivery of copper to Complex IV reaching the complex from the IMS [43], [53]. yCoa6 is also required for efficient formation of respiratory supercomplexes comprised of Complexes III and IV [43]. Its transcription is induced in response to specific DNA-damaging agents and the protein abundance increases in response to DNA replication stress [54], [55]. Taken together, the data suggest that this protein may be involved in stress response and respiration. The detection of human Coa7b, a complex IV assembly factor is therefore not surprising. Detection of yQcr6 (subunit 6 of the ubiquinol cytochrome-c reductase complex) in the IMS seemed to be less unexpected. This subunit is discussed by Schmitt et al., Yang et al., and Brandt [56], [57], [58]. This highly acidic protein is required for maturation of cytochrome c_1_ and might be loosely associated with the complex since it is easily released into the intermembrane space according to these studies and would therefore account for its detection in the IMS. yPet191 characterised by McEwen, et al., is described as essential for cytochrome c oxidase maturation in recent literature [47], [59]. yCox11 is anchored to the mitochondrial inner membrane by a single transmembrane segment, whereas its C terminus contains a copper-binding domain, is exposed to the intermembrane space and forms a homodimer that binds two Cu(I) ions. Copper is delivered to yCox11 by yCox17 [60]. Also, subunit VIb of human complex IV (Cox12 in yeast) is described as only loosely attached to the respiratory complex IV and allows detection of this subunit without surprise in the IMS [61].

The *Saccharomyces cerevisiae* RIB4 gene codes for 6,7-dimethyl-8-ribityllumazine synthase involved in riboflavin biosynthesis [62]. Vögtle et al. discuss it as a putative candidate required for respiration similar to its homolog yRIB3 [43], [63].

Aras and co-workers suggested the renaming of the hCHCHD2 (AAG10, C7orf17) protein as mitochondria nuclear retrograde regulator 1 (hMNRR1) [64]. They confirm the IMS localisation and its import as being Mia40 dependent. Its presence and association with complex IV in the IMS is required for COX activity. Upon stress, hMNRR1 localizes to the nucleus and acts as transcription factor. One of its targets is the hCOX4-2 gene, whose transcription is stimulated in hypoxic conditions. The reported interactome of the hCHCHD2 protein displays candidates such as hCOX5B, a cytochrome c oxidase subunit, hSIRT1 and hHTT (Huntington disease protein homolog). hSIRT1 was shown to de-acetylate and affect the activity of both members of the PGC1-alpha/ERR-alpha complex, which are essential metabolic regulatory transcription factors and are known to regulate expression of respiratory chain complex subunits. Complex IV is the most affected respiratory chain complex in Huntington's disease. Taking these facts together, it is tempting to ask the question if hCHCHD2 takes part in the communication path between respiratory chain assembly and PGC1-mediated signalling. Interestingly, Liu et al. discuss hCHCHD2 as one of the negative regulators of mitochondria-mediated apoptosis with no known homology to the Bcl-2 family, while Singleton detected mutations in the hCHCHD2 gene of late onset Parkinson patients [65], [66]. Furthermore, Wei and co-authors expand the role of this protein towards cell migration regulation and cancer [67]. This protein is therefore one of the candidates which supports the underestimated role of the IMS proteome as being involved in a complex communication system within the cell.

## 9 IMS candidates in redox homeostasis

The thioredoxin system together with the glutathione/glutaredoxin system help to maintain the reduced cell environment and play a significant role in defending the cell against oxidative stress. The yeast cytoplasmic thioredoxin system is comprised of the thioredoxins yTrx1 and yTrx2 and the thioredoxin reductase yTrr1. The mitochondrial matrix thioredoxin system, on the other hand, is composed of thioredoxin yTrx3 and the thioredoxin reductase yTrr2 [68]. It is speculated that the cytoplasmic thioredoxin system may have an overlapping function with the glutathione/glutaredoxin system, but the mitochondrial thioredoxin system does not appear to be able to substitute for either the cytoplasmic thioredoxin or glutathione/glutaredoxin systems [69], [70]. It seems more likely that the mitochondrial thioredoxin proteins are implicated in oxidative stress defense generated during respiratory metabolism [71]. Assigning the previously only cytosolic proteins yTrr1 and yTrx1 to the IMS will open up the chance to unravel the speculated overlap of the systems or a possible communication path between the two systems. Recent work by Greetham et al. shows evidence that under oxidative stress the redox state of a mitochondrial thioredoxin regulates yeast apoptosis. This finding connects the thioredoxin system with a signalling pathway, which mediates antioxidant defense and cell death at the same time [72].

Support for a redox maintenance system in the IMS is the discovery of Endoplasmic reticulum resident protein 18 (hERp18) in the human IMS orphan data set. This protein belongs to the thioredoxin superfamily, which catalyzes disulfide bond formation and isomerization. This protein is discussed to play a role in the defense against endoplasmic reticulum stress. Equally, it may maintain this function in the IMS [73]. Another candidate for a stress defense mechanism is microsomal glutathione S-transferase 3, also found for the first time in mitochondria. MGST3 has been found to have LTC_4_ (leukotriene-C_4_) synthase activity and is able to reduce 5-HPETE ((S)-5-hydroperoxy-8,11,14-*cis*-6-*trans*-eicosatetraenoic acid) through the utilisation of GSH [74].

yHyr1 (also known as Gpx3 or Orp1) is a thiol peroxidase which functions as a hydroperoxide receptor to sense intracellular hydroperoxide levels and transduce a redox signal to the yYap1 transcription factor. yHyr1 interacts with the C-terminus of yYap1 via a disulfide bond. Nuclear export of yYap1 is inhibited, resulting in activated yYap1 transcription [75]. The Hyr1-dependent activation of yYap1 is also dependent on the protein yYbp1 [76]. Localisation of a transcription factor interacting protein to the IMS might allow us to investigate mechanisms which regulate transcription after receiving a mitochondrial signal.

The proteins encoded by the hPRDX3 and hPRDX4 genes are antioxidant enzymes belonging to the peroxiredoxin family. The hPRDX4 protein was localized to the cytoplasm. Peroxidases of the peroxiredoxin family reduce hydrogen peroxide and alkyl hydroperoxides to water and alcohol with participation of reducing equivalents derived from thiol-containing donor molecules. The presence of these proteins in a subcellular compartment with a more oxidative milieu points towards a specific function of the IMS in the pathway of oxidative stress response and make both proteins highly interesting candidates to investigate oxidative stress signalling [77], [78].

## 10 IMS candidates for apoptosis signalling

yHmf1 is a member of the p14.5 protein family and is heat shock inducible. It was shown to functionally complement yMmf1 when targeted to mitochondria. Oxelmark et al. identified yMmf1 as a novel yeast mitochondrial protein involved in maintenance of the mitochondrial genome, whereas members of the 14.5 protein family are often involved in apoptotic pathways [79]. Therefore, it might be interesting to investigate yHmf1's specific function and the connection of mitochondrial genome maintenance in context of apoptosis signalling. hFAM162A (C3orf28) is proposed to be involved in regulation of apoptosis. The exact mechanism may differ between cell types/tissues. hFAM162A is speculated to be involved in hypoxia-induced cell death with cytochrome *c* release and caspase activation and inducing mitochondrial permeability transition. In neuronal cells, hFAM162A might promote release of hAIFM1 from mitochondria to the cytoplasm followed by its translocation to the nucleus; however, the involvement of caspases has been debated upon in the literature [80], [81]. The protein interacts with hHSP90AB, which is essential for hFAM162A mitochondrial localization and pro-apoptotic activity. The reported interaction with hVDAC2 explains the link in inducing the mitochondrial permeability transition.

## 11 IMS candidates for lipid signalling

An interconnected and truly complex signalling network which modulates a variety of cell events is regulated by production versus elimination of signalling lipids such as e.g. phosphatidic acid (PA), diacylglycerol (DAG), and phosphatidylinositol 4,5-bisphosphate (PI4,5P2). PA can be generated by hydrolysis of cardiolipin mediated by e.g. a newly identified, non-canonical member of the PLD superfamily named MitoPLD, which localizes to the mitochondrial surface and participates in mitochondrial fusion. In addition, PA on the mitochondrial surface may also trigger a signalling cascade that elevates DAG, leading to downstream events that affect mitochondrial fission and energy production or may stimulate local production of PI4,5P2 to facilitate subcellular trafficking or Ca^2 +^ influx [82]. Identification of proteins involved in lipid metabolism, not only at the mitochondrial envelope but also in the IMS, might therefore unravel necessary links to mediate signalling from the surface to the matrix or vice versa. In this context, further investigation will be required as to why enzymes of the beta-oxidation machinery are needed in the IMS.

One of these candidate proteins is yPot1, which is also known to be present in peroxisomes. yPot1, a 3-ketoacyl-CoA thiolase with broad chain length specificity, cleaves 3-ketoacyl-CoA into acyl-CoA and acetyl-CoA during beta-oxidation of fatty acids.

yPdx3 pyridoxine (pyridoxamine) phosphate oxidase, instead, is reported from Loubbardi et al. to be involved in sterol uptake, with the mutant yeast strain displaying atypical fatty acid, sterol, and cytochrome patterns [83]. Detecting components of lipid metabolism regulation in the IMS contributes either to the general homeostasis of this sub-compartment or harbours the potential to investigate new routes of lipid signalling.

The transport of phospholipids to mitochondrial membranes remains unclear, except for the fact that the endoplasmic reticulum (ER) contact sites might be involved [84]. In this context, it is important to note that the component of the MICOS complex hMIC26 was detected in the human set of IMS orphans. This protein is not only involved in cristae formation by modulating cristae junctions but has also been reported to play a role in lipid transport [85], [86]. Furthermore, the traffic of phospholipids between the mitochondrial membranes is far from being understood. Recently, Connerth et al. were able to identify yUps1 as phosphatic acid shuttle protein, residing in the IMS [87]. hACP6 protein, one of the recent orphans, hydrolyses lysophosphatidic acid (LPA), containing a medium length fatty acid chain to the corresponding monoacylglycerol. Hiroyama and Takenawa propose involvement of ACP6 in mitochondrial lipid biosynthesis regulation [88]. Also, hGTT1, a mitochondrial StAR-related lipid transfer protein 7 with the ability to transfer phophatidylcholine [89], extends the list of IMS proteins involved in lipid signalling. hSERAC1 (serin active site containing) is a phosphatidylglycerol remodeling protein that has so far been found at the interface between the mitochondria and the ER, where it mediates phospholipid exchange. The encoded protein plays a major role in mitochondrial function and intracellular cholesterol trafficking. Defects in this gene are a cause of 3-methylglutaconic aciduria with deafness, encephalopathy, and Leigh-like syndrome (MEGDEL) [90]. Therefore, the assignment of this protein to the IMS might highlight a regulatory cycle of controlled phospholipid exchange. The protein encoded by the hAGK gene is a mitochondrial membrane protein involved in lipid and glycerolipid metabolism. The lipid kinase catalyzes the formation of phosphatidic and lysophosphatidic acids. Defects in this gene have been associated with mitochondrial DNA depletion syndrome 10 [91]. To summarize, the assignment of candidates being involved in lipid metabolism highlight the need to investigate to what extent the IMS contributes to lipid signalling pathways.

## 12 IMS candidates for calcium homeostasis, Alzheimer's disease and beyond

Orphans which add even more diversity to pathways, but have not been investigated in detail in the context of mitochondrial research are discussed below.

The discovery of two different SNARE proteins hSTX17 and hSNAP29 are a surprise but, in the context of mitophagy processes, they are important to consider.

yMtf1 is a mitochondrial RNA polymerase specificity factor [92] displaying structural similarity to S-adenosylmethionine-dependent methyltransferases. yMtf1 interacts with the Rpo41-promoter complex and stabilizes the complex, thusenhancing DNA bending and melting to facilitate the pre-initiation open complex formation. This particular protein was also found to be required for promoter specific activity through the suppression of Rpo1 non-specific transcription. [93]. The yMpm1 protein is an intermembrane space protein with unknown function. Therefore, interpretation of its reported interactome revealed interaction with a group of proteins involved in cristae formation and membrane organisation such as yMic10, yMic12, yMic60 and yMdm10. More exciting is the evidence for interaction of yMpm1 with yMpc1, a highly conserved subunit of the mitochondrial pyruvate carrier. This carrier is located in the mitochondrial inner membrane and is comprised of yMpc1 and yMpc2 or yMpc3. Mutations in yMpc1 deplete pyruvate uptake in mitochondria [94]. The assignment of yMpm1 to the IMS putatively interacting with the pyruvate carrier of the inner membrane might be interesting to explore, since regulation of the pyruvate carrier is far from understood but harbours potential for cancer therapy [95].

IMS candidates with dual localisation which are candidates for further investigation can be found in Table 1, with its listing of localisation to other cell compartments. Less interesting candidates are yGpm1, which is involved in glycolysis, and Ybr056w, a putative glycoside hydrolase of the mitochondrial intermembrane space. A yNCE103 deletion was reported by Götz et al., producing an oxygen sensitive phenotype with reduced growth under aerobic conditions [96].

The association of a prostaglandin E synthase named hPTGES2 (C9orf15) with the IMS raises interest at a second glance when discovering the reported literature [97], which speculates a function for this IMS protein in replacing the cytosolic PGES in later stages of Alzheimer's disease.

The full-length protein encoded by the hHEBP1 gene is an intracellular tetrapyrrole-binding protein. This protein includes a natural chemoattractant peptide of 21 amino acids at the N-terminus, which is a natural ligand for formyl peptide receptor-like receptor 2 (FPRL2) and promotes calcium mobilization and chemotaxis in monocytes and dendritic cells. A fair question to be asked would be if this protein participates in calcium regulation in the IMS. Another orphan putatively involved in calcium signalling is hPPIF, the human homolog of yCpr1. In a study using mice as a model system, the importance of this protein and its involvement in the permeability transition pore, thereby regulating calcium levels, are investigated [98]. Regulation of Ca^2 +^ levels in mitochondria is a critically important task. The involvement of the IMS in this particular process is under investigation and gains further support from the discovery of two proteins which might be involved: Calcium-binding protein hERC-55 [99] and hNEFA [100]. hERC-55 even offers the possibility to be a player in the redox defense pathways, since it is shown to interact with peroxiredoxin-6, too. Therefore, it becomes a highly interesting candidate to investigate.

## 13 Cross-talk mediated from metabolites and epigenetics

One of the exciting questions of recent research is how metabolism integrates with epigenetic and genetic programs to regulate cell function and especially pluripotent stem cell (PSC) fate and function. The regulation of metabolite fluxes is achieved by transcription factors including c-Myc and HIF1α and signalling molecules such as PI3K, AKT, and mTOR [101]. Studies in mammalian cells have shown that metabolites can function as cofactors or substrates for chromatin structure regulating enzymes and influence gene expression. The role of mitochondria, hosting several biosynthetic pathways and being a source of signalling ROS, will be important to dissect in the future. Especially in the field of stem cell research, the understanding of the role of mitochondria in the context of metabolite and signalling cross-talk will prove to be very exciting. Acetyl-CoA generated in diverse pathways of glucose, fatty acid, or amino acid catabolism in mitochondria can be transported to the cytosol and be targeted to the nucleus to increase histone acetylation [102], [103]. Another mitochondrial metabolite, α-ketoglutarate, can exit the organelle to serve as a cofactor for dioxygenase enzymes including TET-family DNA hydroxylases, Jumonji-family histone demethylases and, eventually, prolyl hydroxylases which themselves control HIF1α/2α transcription factor stability [104].

Candidates that are discussed to have the potential to be located in the IMS but might be targeted there with transient localisation in the nucleus could be relevant to explore the communication and regulation of gene expression. The orphan yHmf1, as well as yCpr1, are such candidates. hCiapin1 (yDre2) displays features of typical IMS proteins, such as the cysteine motif in combination with the hydrophobic stretch [105]. Additionally, the flexible C-terminus resembles a methyltransferase motif. The protein has been reported to be present in the nucleus, [106], [107] and has been found on the mitochondrial surface. Such a localisation would be compatible with a role in translating signals from mitochondria to the nucleus thereby influencing gene expression. Research with cancer cells has shown that defects in TCA cycle enzymes, such as succinate dehydrogenase, fumarate hydratase, and isocitrate dehydrogenase, cause inherited benign or malignant tumours by altering DNA and histone modifications thereby causing transcriptional dysregulation [108].

Deacetylase enzymes, such as the Sirtuin family proteins, are sensitive to the redox state of the cell compartment and can impact histone modifications and posttranscriptional changes through non-histone protein deacetylation [109]. Many biological processes are regulated by metabolic activities and/or are impacted by the cellular redox state. For example, the redox state modulates the balance between self-renewal versus differentiation in dividing glial precursor cells [110]. The accumulation of ROS is minimized in PSCs, a feat which is achieved by a reduction in substrate oxidation and respiratory coupling. It is still under investigation how the transition from uncoupled to coupled respiration during differentiation is achieved. The detection of proteins like hCHCHD2 (C7orf17), which might interact with PGC1-alpha, in the IMS, make them attractive candidates to investigate.

Additionally, the elevated expression of antioxidant stress genes such as yUCP2, ySOD2, and yGPX2 contribute to the lower ROS level. The assignment of yHyr1 to the yeast intermembrane space extends the list of enzymes which might mediate response to ROS specifically in the IMS. As soon as the differentiation process is induced, these genes become repressed [111], [112]. Increase of ROS promotes lineage-specific differentiation, which in the case of cardiomyocyte precursor cells is mediated by induction of the p38 MAPK stress signalling pathway [113]. In the case of cancer cells, the effect of increased ROS accumulation promotes cell proliferation but not differentiation [114]. The mechanisms for this differential response to ROS in distinct cellular contexts will require further investigation and are exciting complementations to mitochondrial research. During mouse Embryonic Stem Cell (mESC) differentiation, the ratio of reduced to oxidized glutathione decreases as well as the level of NADH, while the unsaturated metabolome with carbon-carbon double bonds is replaced by an oxidative metabolome, effectively increasing the overall cellular oxidation state [115], [116]. Yanes and coworkers demonstrated that blocking the oxidative eicosanoid signalling cascade in ESCs can also delay differentiation [116]. Tight regulation of specific redox species, such as the NAD +/NADH ratio, is critical for signalling events that require NAD +-dependent deacetylase activities [117]. Since the NAD +/NADH ratio is regulated by glycolytic and mitochondrial activities that change dramatically during differentiation or reprogramming [118], the NAD +/NADH redox state may have a role in driving PSC fate. These interesting findings linked to stem cell research inversely stimulate the discussion of so far underestimated regulations of the redox state in the different cell compartments due to their different oxidative milieu. Investigating new IMS orphans which might be involved in redox state homeostasis, such as yTrx1 and yTrr1 with their dual localisation, therefore sparks new fields for mitochondrial research.

## 14 Future directions

**Fig. 1:**
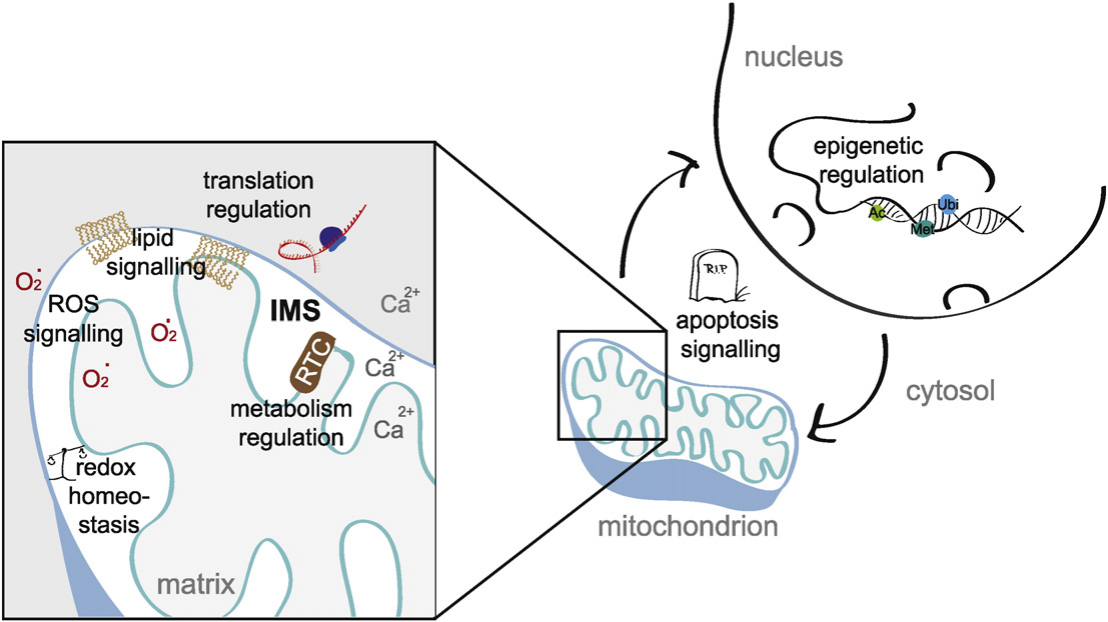
The IMS proteome of mitochondria is involved in several cellular processes. These include (i) epigenetic regulation in the nucleus, (ii) apoptosis signalling, (iii) regulation of translation, (iv) lipid signalling, (v) signalling via reactive oxygen species (ROS), (vi) maintenance of redox homeostasis and (vii) regulation of metabolism and of the respiratory transport chain (RTC).

Here, we have discussed the current knowledge and function of proteins which were only recently assigned to the IMS. The novel orphans were investigated with prediction tools to allow a first indication on which function this protein might be involved in. Also, information from protein databases and previous literature has been interpreted to spark speculation about future research. In this review, we have highlighted fragmented evidence of proteins which have been newly assigned to the IMS and have tried to integrate this information together with functional data and analysis of pathways that may help us to unravel some of the future challenging questions on how mitochondria have a central role in several signalling processes in the cell (depicted schematically in Fig. 1).

Based on findings related to cancer or stem cell research we can aim to unmask the communication mechanisms between the cell organelles, specifically between other compartments and mitochondria. It is also important to note that we need to integrate the knowledge of the metabolome, the metabolism and its regulation with the proteome and traditional signalling pathways to broaden our understanding and start the journey of exploring the next step in mitochondrial research. Here, we have featured the main traditional knowledge about mitochondria, signalling and protein sorting and tried to incorporate the knowledge which was discovered in cancer and stem cell science. We have focused especially on what we can learn from the newly identified orphans of the IMS in the context of intracellular communication. We have found links to apoptosis, lipid, metal and redox stress signalling. Future projects might focus specifically on addressing some of these signalling pathways, which are mediated by the new identified IMS orphans, and help us to understand how mitochondria contribute to the whole physiology of a healthy and functional cell.

## Conflict of interest

The authors declare no conflict of interests or any commercial associations.

## Figures and Tables

**Table 1:** Summary of the newly identified protein members of the intermembrane space proteome. Assignment of gene names and accession numbers was achieved by following annotations from UniProt/SGD. Cell compartment annotation as summarized in the table is based on evidence found in specific literature. The “Yeast/Human homolog” column refers to the homologous yeast proteins in the case of the human proteome and the homologous human proteins in the case of the yeast proteome. The “Mitochondrial/IMS localisation verified” column refers to the proteins that have been verified experimentally (western blotting or fluorescence microscopy) to have a mitochondrial localisation for the human proteome or an IMS specific localisation for the yeast proteome. (* Laminin contains a very large number of both typical (eg. CX_9_C) and non-typical (eg. CX_5_C) cysteine motifs).

UniProt accession number	Protein names	Gene names	Yeast/ human homolog	Molecular weight (kDa)	CX_n_C motif	Previous cell compartment annotation	Mitochondrial/ IMS localisation verified?
**Human proteome**							
Q9NUJ1	Mycophenolic acid acyl-glucuronide esterase, mitochondrial	ABHD10	–	33.9	CX_6_C, CX_9_C	–	No
Q9NPH0	Lysophosphatidic acid phosphatase type 6	ACP6	–	48.9	CX_7_C, CX_4_C	Mitochondria [1]	No
Q53H12	Acylglycerol kinase, mitochondrial	AGK, MULK	–	47.1	CX_13_CX_8_C, CX_5_C	Mitochondrial membrane [2]	No
Q9BUR5	MICOS complex subunit MIC26	APOO, MIC23, MIC26	–	22.3	CX_6_C	ER/Golgi, inner mitochondrial membrane [3]	Yes [4]
Q96BQ5	Coiled-coil domain-containing protein 127	CCDC127	–	30.8	–	–	Yes [4]
Q4VC31	Coiled-coil domain-containing protein 58	CCDC58	–	16.6	–	–	No
Q4G0I0	Protein CCSMST1	CCSMST1	–	15.0	CX_8_C	–	No
P30307	M-phase inducer phosphatase 3	CDC25C	–	53.4	CX_6_CX_16_C, CX_7_C, CX_10_CXC, CX_5_CX_13_C	Nucleus [5]	Yes [4]
A8MTT3	Protein CEBPZOS	CEBPZOS	–	9.3	–	–	Yes [4]
Q9H078-2	Caseinolytic peptidase B protein homolog	CLPB, HSP78, SKD3	–	75.4	CX_9_C	Mitochondria [6]	No
Q9NRP2	COX assembly mitochondrial protein 2 homolog	CMC2, C16orf61	CMC2	9.5	Twin CX_9_C	Inner membrane facing IMS [7]	No
Q5JTJ3-2	Cytochrome c oxidase assembly factor 6 homolog	COA6, C1orf31	COA6	14.1	CX_12_C, CX_9_CX_10_CX_11_C	–	Yes [8]
Q96BR5	Cytochrome c oxidase assembly factor 7	COA7, RESA1	–	25.7	CX_3_CX_8_C, CX_8_C, CX_4_CX_10_C, CX_7_C, CX_6_CX_7_C	–	Yes [4,9]
Q9P0S2	Cytochrome c oxidase assembly protein COX16 homolog, mitochondrial	COX16, C14orf112	COX16	12.3	–	Mitochondrial membrane [10]	No
Q5RI15	Cytochrome c oxidase protein 20 homolog	COX20, FAM36A	COX20	13.3	CX_13_CX_3_C	–	No
P36957	Dihydrolipoyllysine-residue succinyltransferase component of 2-oxoglutarate dehydrogenase complex, mitochondrial	DLST, DLTS	KGD2	48.8	CX_16_CX_12_C	–	No
E7EQY1	Protein FAM136A	FAM136A	–	26.8	CX_9_C, CX_3_CC, triple CX_3_C	–	No
Q96A26	Protein FAM162A	FAM162A, C3orf28	–	17.3	CX_3_C	–	No
Q96AY3	Peptidyl-prolyl cis-trans isomerase FKBP10	FKBP10	FPR2	64.2	CX_15_C	–	Yes [4]
Q9NRV9	Heme-binding protein 1	HEBP1, HBP	–	21.1	–	Cytosol [11]	No
P11047	Laminin subunit gamma-1	LAMC1	–	177.6	CX_n_C*	–	No
O14880	Microsomal glutathione S-transferase 3	MGST3	–	16.5	–	–	Yes [4]
Q9Y6H1	Coiled-coil-helix-coiled-coil-helix domain-containing protein 2, mitochondrial; Aging-associated gene 10 protein	MNRR1, CHCHD2, AAG10, C7orf17	MIX17	15.5	CX_9_CX_9_CX_9_C	Nucleus [12], mitochondria [13]	No
Q14596	Next to BRCA1 gene 1 protein	NBR1	–	107.4	CX_2_CX_10_CX_2_CX_5_CX_2_C	Cytosol [14]	Yes [4]
P80303	Nucleobindin-2	NUCB2, NEFA	–	50.2	CX_5_C	Golgi apparatus [15]	No
Q9NX40	OCIA domain-containing protein 1, Ovarian carcinoma immunoreactive antigen	OCIAD1, OCIA	–	27.6	CX_14_C	–	No
P07237	Protein disulfide-isomerase	P4HB	PDI1	57.1	Twin CX_2_C	ER [16,17]	No
Q9H7Z7	Prostaglandin E synthase 2	PGES2, C9orf15	–	41.9	CX_2_C	Golgi apparatus membrane [18]	No
P30048	Thioredoxin-dependent peroxide reductase, mitochondrial	PRDX3, AOP1	TSA1	27.7	CX_9_CX_14_C	–	Yes [19]
Q13162	Peroxiredoxin-4	PRDX4	TSA1	30.5	–	Cytosol [20]	No
Q14257	Reticulocalbin-2	RCN2, ERC55	–	36.9	–	–	No
Q96DB5	Regulator of microtubule dynamics protein 1	RMDN1, FAM82B	–	35.8	CX_2_C	–	No
Q96JX3	Protein SERAC1	SERAC1	–	74.1	CX_2_CC, CX_13_C, CX_9_C	Mitochondria-ER interface [21]	No
O95721	Synaptosomal-associated protein 29	SNAP29	–	29.0	–	Cytosol [22]	No
Q9NQZ5	StAR-related lipid transfer protein 7, mitochondrial	STARD7, GTT1	–	43.1	–	–	No
P56962	Syntaxin-17	STX17	–	33.4	CX_10_C	ER, ERGIC [23]	Yes [4]
O95881	Thioredoxin domain-containing protein 12, ERp18	TXNDC12	–	19.2	CX_2_C	ER [24]	No
**Yeast proteome**							
Q3E846	Cytochrome c oxidase assembly factor 6	COA6 (YMR244C-A)	COA6	12.4	CX_9_C, CX_10_C	–	Yes [8,25]
P19516	Cytochrome c oxidase assembly protein COX11, mitochondrial	COX11 (YPL132W)	COX11	34.0	CXC	Mitochondrial membrane [26]	No
Q01519	Subunit VIB of cytochrome c oxidase	COX12 (YLR038C)	COX6B2	9.8	CX_9_C, CX_10_C	Integral to IM [27]	Yes [25]
P14832	Cytoplasmic peptidyl-prolyl cis-trans isomerase	CPR1 (YDR155C)	PPIF	17.4	–	Cytosol [28]	Yes [25]
P00950	Tetrameric phosphoglycerate mutase	GPM1 (YKL152C)	PGAM2	27.6	–	Cytosol, extracellular [29]	Yes [25]
P40037	Homologous Mmf1p Factor , member of the p14.5 protein family	HMF1 (YER057C)	–	13.9	–	Cytosol [30]	Yes [25]
P40581	Peroxiredoxin HYR1	HYR1 (YIR037W)	–	18.6	–	Cytosol [31]	Yes [25]
P38162	Mitochondrial intermembrane space cysteine motif-containing protein MIX23	MIX23 (YBL107C)	–	23.0	CX_14_C, CX_13_C	–	Yes [25]
P40364	Mitochondrial peculiar membrane protein 1	MPM1 (YJL066C)	–	28.5	–	Mitochondrial membrane [32]	Yes [25]
P14908	Mitochondrial transcription factor 1	MTF1 (YMR228W)	–	39.7	–	Mitochondria [33,34]	No
P53615	Carbonic anhydrase	NCE103 (YNL036W)	–	24.9	CX_8_C, CX_16_CX_8_CX_4_CX_5_CX_12_C	–	Yes [25]
P38075	Pyridoxine phosphate oxidase	PDX3 (YBR035C)	PNPO	26.9	–	–	No
Q02772	Protein required for assembly of cytochrome c oxidase	PET191 (YJR034W)	–	12.4	Twin CX_9_C	Integral to IM facing the IMS [35]	Yes [25]
P27796	3-ketoacyl-CoA thiolase	POT1 (YIL160C)	ACAA1	44.7	CX_16_C	Peroxisome [36]	No
Q12511	Mitochondrial type 2C protein phosphatase	PTC5 (YOR090C)	PDP1	63.7	–	–	Yes [25]
P00127	Cytochrome b-c1 complex subunit 6	QCR6 (YFR033C)	–	17.3	–	Mitochondrial membrane [37,38]	Yes [25]
P50861	Lumazine synthase	RIB4 (YOL143C)	–	18.6	–	–	Yes [25]
P29509	Cytoplasmic thioredoxin reductase	TRR1 (YDR353W)	–	34.2	CX_2_C	Cytosol [39]	Yes [25]
P22217	Cytoplasmic thioredoxin	TRX1 (YLR043C)	TXN	11.2	CX_2_C	Cytosol [39]	Yes [25]
P38081	Putative glycoside hydrolase of the mitochondrial intermembrane space	YBR056W	–	57.8	–	–	No
